# Associations between welding fume exposure and blood hemostatic parameters among workers exposed to welding fumes in confined space in Chonburi, Thailand

**DOI:** 10.1371/journal.pone.0260065

**Published:** 2021-11-18

**Authors:** Ning Li, Nutta Taneepanichskul

**Affiliations:** 1 College of Public Health Sciences, Chulalongkorn University, Bangkok, Thailand; 2 Key Laboratory of Radiological Protection and Nuclear Emergency, National Institute for Radiological Protection, Chinese Centre for Disease Control and Prevention, Beijing, China; 3 Faculty of Medicine, HAUS IAQ Research Unit, Department of Pediatrics, Chulalongkorn University, King Chulalongkorn Memorial Hospital, Bangkok, Thailand; Institute of Health and Society - Federal University of São Paulo, BRAZIL

## Abstract

**Background:**

Occupational welding fumes contain varieties of toxic metal particles and may affect cardiovascular system like the Particulate Matters (PM). Few studies have focused on the effects of toxic metals on the hemodynamic balance; however, the reporting results were not consistent. This study aimed to investigate the association between toxic metals exposure (Chromium (Cr), Manganese (Mn) and Lead (Pb)) and blood hemostatic parameters status after a 3-week exposure cessation among workers exposed to welding fumes.

**Methodology:**

Structured interviews and biological samplings were conducted for 86 male workers without a history of Anemia and Cardiovascular diseases (CVDs) and working in a confined space to construct crude oil tanks. Metal levels of Cr, Mn and Pb in urine were measured during the working days using Inductively Coupled Plasma Mass Spectrometer (ICP-MS) method. The concentrations of hemostatic proteins in blood (White blood cell counts (WBC), Lymphocytes, Monocyte, Eosinophil, Neutrophil, Hematocrit (Hct) were assessed after a 3 weeks exposure cessation. Workers were divided into groups based on occupation type (welder group and non-welder group), and based on metal levels (high and low exposure groups) for comparison. Linear regression models were used to explore the association between metal exposure and multiple blood hemostatic parameters adjusted for age, Body Mass Index (BMI), and smoking status.

**Results:**

Urine Mn and Cr level of the welder group was significantly higher than the non-welder group (Mn: 0.96 VS 0.22 ug/g creatinine, p < 0.001; Cr: 0.63 VS 0.22 ug/g creatinine, p < 0.01). The mean value of Hct in the welder group was 44.58 ± 2.84 vol%, significantly higher than the non-welder group (43.07 ± 3.31 vol%, p = 0.026). The median value of WBC in the high Mn-exposed group (6.93 ± 1.59 X 10^6^ Cell/ml) was significantly lower than the low Mn-exposed group (7.90 ± 2.13 X 10^6^ Cell/ml, p = 0.018). The linear regression analyses showed that there was a significantly negative association between log transformed WBC value and the Mn exposure groups (high and low) after adjusting for age, BMI, and smoking status (β = - 0.049, p = 0.045), but no significant result was found between WBC and occupation types (welder and non-welder) (p > 0.05). Multiple linear regression analysis also showed positive association between Hct and occupational types (welder and non-welders) (β = 0.014, p = 0.055). The other hemostatic parameters were not different from controls when divided by occupation type or metal level groups.

**Conclusions:**

Our results showed that welders were exposed to about 3 to 4 times higher Mn and Cr concentrations than non-welders. Moreover, one third of the non-welders were exposed to high-exposure groups of Mn and Cr metals. Regression models revealed a significant association of the WBC counts with the Mn exposure group. Therefore, we infer that Mn exposure may play a significant role on the blood hemostatic parameters of workers in the confined space. Hazard identification for non-welders should also be conducted in the confined space.

## Instruction

Toxic metal pollution has become a growing public health concern with its potential to cause cardiovascular diseases resulting from increasing industrialization and its associated activities [1–3]. Welders are exposed to acute and long-term occupational welding fumes with metal particles, which are assumed to be more harmful than the normal ambient PM_2.5_ exposure [4]. Hypothetical mechanisms of welding fume toxicity center on oxidative stress and inflammatory reactions, similar to the mechanism that link PM_2.5_ and cardiovascular diseases (CVDs) [5–7]. The popular hypothesis that has been proposed is that particles and metals can initiate an acute and chronic inflammatory response and act as a source of local irritation because of their physical, chemical, or biological properties, and thus, inhaled particles cause inflammation in the lungs and a series of systemic inflammatory reactions due to the circulation of metals in the blood [5,8]. The metals in welding fumes can cause an imbalance between the production and detoxification of reactive oxygen species (ROS), which may promote oxidation of amino acids in proteins, lipid peroxidation, cell membrane damage and DNA damage, and other metal-related, ROS-mediated consequences [7,9]. A group of cells such as monocytes, T cells, and platelets are formed in response to inflammation. Continued inflammation leads to an increase in the number of macrophages and lymphocytes, both of which can emigrate from the bloodstream and multiply during injury within the process of lesion [10]. The pro-oxidation and pro-inflammatory effects on multiple organs, tissues, cells, and various molecular mediators can enhance the process of atherosclerosis, followed by other cardiovascular events, which are the most common pathological processes leading to CVDs [5,11].

In recent years, studies have shown that short-term or long-term exposure to air pollutants may affect hemodynamic balance. In short, existing evidences have shown that various pro-oxidant, inflammatory and/or hemodynamic agents can "spill" into the systemic circulation triggering a range of other related reactions, such as cytokines, activated white blood cells and platelets, oxidized lipids [5,12,13]. Descriptions of changes in hematocrit (Hct), Hemoglobin, WBC, Red blood cells, blood neutrophils, platelets fibrinogen [14–17], have suggested that biological effects of particle inhalation may also affect the vascular system. The Hct, also known by several other names such as packed cell volume (PCV), volume of packed red cells (VPRC), is the volume percentage (vol%) of red blood cells in blood and is the point of reference of its capability of delivering oxygen. An abnormally low hematocrit may suggest anemia, a decrease in the total amount of red blood cells, while an abnormally high hematocrit is called polycythemia [18]. White blood cells (WBC) and its difference, in particular neutrophils, are the primary acute inflammatory cells. Inflammation may also promote atherosclerotic plaque rupture and thrombosis. Leukocytes or WBC may serve as an important biomarker for these disease processes and it was reported that elevated WBC may also be a risk factor for acute myocardial infarction, coronary artery disease, coronary heart disease (CHD), and stroke [19,20].

The process of welding metals may lead to substantial exposure to particles, fumes, and gases. The size of aero-disperse toxic particles varies from 0.005μm to 20mm, commonly referred to as welding fume. There are 13 metals currently known to be present in welding fumes including chromium (Cr), cobalt (Co), zinc (Zn), lead (Pb), iron (Fe), cadmium (Cd), beryllium (Be), mercury (Hg), and Mn [21,22]. Most of these metals have shown to be both cytotoxic and neurotoxic [22,23]. The fumes generated during mild steel welding contain mainly Fe (80–95%) and Mn (1–15%), and these two metals have been found to be strongly correlated. On the other hand, stainless steel welding produces higher amounts of Cr (15–30%) and nickel (5–10%), which are also highly correlated [24]. In this study, the workers performed Flux Core-Arc Welding (FCAW) and Submerged-Arc Welding (SAW) for welding of piping and crude oil tanks. The bare steel of the base, or the storage tank, is mainly made of carbon-manganese-silicon steel (mild steel).

There are more and more workers worldwide performing welding as a part of their job with the urban civilization, especially in developing counties like Thailand. However, not many articles were found to have reported about the health effects of occupational exposure to toxic metals in confined spaces. Previous studies reported inconsistent results for the effects of welding fumes exposures on blood parameters. In this study, structured interviews were used to gather information from the participants on a variety of demographic factors, health behaviors (second-hand smoking exposure, alcohol drinking, sleep quality, physical activity), welding exposure conditions, health condition, and exposure characteristics. Urinary metal (Cr, Mn, Pb, μg/g creatinine) level were checked using ICP-MS. Plasma and serum samples were collected from peripheral blood for measurement of WBC, Hct, platelets, monocyte, lymphocyte, and neutrophils. The effects of toxic metals (Mn, Cr and Pb) exposure on blood hematological parameters in welders and non-welders working in confined space were analyzed using linear regression models. Differences between occupation type and metal level groups were investigated to infer the hazard of occupational welding fume exposure on the blood hemostatic parameters.

## Methods

### Study design, population, and welding process

This study was a part of an ongoing longitudinal study designed to study the effect of welding fumes on hemostatic blood parameters and cardiovascular system among pipeline welders and workers working in confined space in Thailand. There were about 250 workers working to construct the crude oil tank near the Laem Chabang Port in Chonburi province, which was the largest deep-sea port and the regional hub for petroleum and petrochemical shipments. The enrolment was carried out in 2018 with eligibility criteria as follows: with certificate of working in confined space; full time male workers with contract more than one year; age between 25 to 50 years; with Thai literacy. There were 175 male workers met the inclusion criteria and exclusion were done as follows: BMI > 28 (20 people), self-reported a history of Diabetes, Hyperlipidaemia, Metabolic syndrome (11 people), or self-reported use of hypertension and CVDs medication (21 people), there were 123 eligible respondents were recruited in this present study at the study starting point. During the following up, 27 people lose follow up, 10 people’s data were cancelled because of incomplete questionnaire and abnormal WBC counts. The study recruited 86 subjects with confined space working certificates, including occupations of welders and non-welders were recruited in the study and followed up. All the participants (both welders and non-welders) worked in tanks in the same working environments, had certificates for working in confined spaces, under conditions where there was relatively efficient local exhaust ventilation. Welders were the full-time occupational welders who conducted the works of welding and connecting the pipes and tanks. Welders were provided with professional helmet and respirators (respirator film 3M 2091 P3-BR) as the Professional Protection Equipment (PPE). Non-welder group contains fitters who put together or installs the steel structure of the tank or pipes, fire watch for taking care the welding machine and some labor work, riggers, painters, and persons working as helper, driver, foreman and quality control.

In the construction process, 9 sets of crude oil storage tanks were constructed, including Dia.58m*Hei.22.3m 4 sets, Dia.83m*Hei.22.3m 5 sets. The storage tanks and other main steel structure components were manufactured by welding all the bare heavy-duty steel and plates together. They primarily performed weld processes for welding of piping and tank were Flux Core-Arc Welding with shielding gas (FCAW) and Submerged-Arc Welding (SAW). The bare steel of the base, or the storage tank, was mainly made of carbon-manganese-silicon steel plate A573 GR70, a steel with standard specification for Structural Carbon Steel Plates of Improved Toughness. The composition properties of A573 GR70 were as follows: Carbon max (0.23%), Mn (0.85–1.2%), Phosphorus max (0.030%), Sulfur max (0.030%), Silicon (0.15–0.40%). In this process, they used Lincoln welding wire, FAMILIARC LH-2000 covered Electrodes (KOBELCO), HYUNDAI cored wire. The chemical composition of deposited metal of E7016, as a low hydrogen, all position electrode used for welding heavy duty steel structures and plates, was like this: Mn (≤1.60%), Si (≤0.75%), P (≤0.040%), Cr (≤0.20%), Ni and Mo (≤0.30%).

### Ethical statement

The study was approved by the Ethics Committee of the Chulalongkorn University Involving Human Research Participants, Health Sciences Group, Chulalongkorn University (RECCU) (COA No.144/2563) and conducted in accordance with the Helsinki Declaration. All study subjects were provided with written informed consent about the objectives and study design prior to participation.

### Questionnaire

All participants finished structured self-report questionnaires administered by the trained research assistant in the working place on the same day as the urine collection. The questionnaire included information on 1) a variety of demographic factors, including age, BMI, education levels, marital status, income; 2) health behaviors, including smoking exposure and passive smoking, alcohol drinking(former & never drinker/ current drinker), sleep quality, diet habit; 3) welding exposure conditions, occupational exposure (yes/no/duration(years)), individual welding history, Personal Protection Equipment (PPEs) use (use respirator, or fabric coverings such as cloth mask and hat), welding type (methods of welding process, Stainless steel and Mild steel welding); 4) health condition (medical history, medicine in sue, family history of CVDs).

### Urine metal analysis

Urine measurement was performed on a selected normal working day when the tank was half completed. All participants agreed to avoid heavy alcohol and heavy exercise within 24 h before urine collection. Middle part of morning urine was taken in a ethylenediaminetetraacetic acid (EDTA) coated test tube, and were transported to the laboratory and stored at—80°C until analysis. The analysis of the monohydroxylated metabolites of pyrene and phenanthrene was performed as described elsewhere with slight modifications. Samples were directly analyzed by ICP-MS with the protocol of previous studies as reference [25,26]. Briefly, urine metal samples were washed by water purification and digested in a 2% nitric solution. Acid digested urine samples were analyzed by using Model 7500a Inductively ICP-MS (Agilent, USA). Limit of detection (LOD) in urine was 0.01 μg/L for Mn and 0.05 μg/L for Cr.

### Blood analysis

Measurements of blood were performed after the day of interview and urine collection about 4 months. Clinical assessment (blood samples) was done before workers returned to work after a 3 weeks work cessation because of the COVID 19 pandemic. All participants agreed to avoid heavy alcohol and heavy exercise within 24 h before blood collection. Blood was collected from the participants via standard venipuncture in a 3 ml EDTA tube. 20 ul of well mixed anti coagulated blood was mixed with 1.98 ml of 3% acetic acid solution that lyses red blood cells and dilutes the blood. The blood-diluent mixture was analyzed using the automated hematology analyzer (Counter HMX Hematology Analyzer) for WBC and differential counting analyses, which including lymphocytes ratio%, Monocyte ratio%, Eosinophil ratio%, Neutrophil ratio%, Hematocrit vol% (Hct vol%). All measurements were performed at the Department of Clinical Chemistry in BMC plus hospital in Chonburi province, and followed the Complete Blood Count (CBC) protocol from the National Health and Nutrition Examination Survey (NHANES) of American CDC [27].

### Statistical analysis

Urine Cr and Mn level below the limits of quantitation (LOQs) were imputed as the test limit value (imputed as 0.10 μg/g creatinine for Cr and Mn, all Pb was above LOQs). Test of normality was performed using the Shapiro-Wilk test. Independent t-test was used for normally distributed continuous data and Chi-square was for categorical data. The Mann Whitney U test was performed for statistical comparisons of skewed distributed values between groups. Urine metal concentration showed non-normal distributions. The metal concentrations were categorized into two groups (High: > median, Low: < median) respectively according to their median values (Mn: 0.59 ug/g creatinine; Cr: 0.50 ug/g creatinine; Pb: 10.89 ug/g creatinine). Pearson correlation was used to evaluate the correlation among log-transformed blood parameters and log-transformed metals (Cr, Mn and Pb). Linear regression models were used to examine associations between blood parameter levels and toxic metals groups (High and Low) and occupation types (Welder and Non-welder) at the log transformed scales. The models were adjusted for covariate factors including age (years), BMI (kg/m^2^), smoke status (former/never smoker, current smoker). Those covariates were selected based on evidence of their statistical significance with WBC concentration in the previous research [17,19]. All statistical analyses were completed by using SPSS 22 for windows (IBM SPSS, version 22, Armonk, NY, USA) and statistical significance refers to P<0.05 (two-tailed).

## Results

The basic demographic characteristics of the population are shown in Table 1. The non-exposure group consisted of fitters, rigger, fire watch, helper, quality control and foreman, a total of 39 individuals. The median ages of the workers were 39.00 and 41.00 years, respectively for the welder group and non-welder group. The median time of the cumulative welding fume exposure of the welder group before working in the oil tank was 9.0 years (47 people self-reported). There was no statistical difference for the distribution of BMI, education level, marital status, smoking status, and smoking fume exposure between the groups (all p > 0.05). The participants in the welder group tended to have a higher level of income, less likely to be current drinkers, but were not significantly different.

**Table 1:** Demographic characteristics of the welding fume exposed workers (n = 86).

Variables	Total (n = 86)	Welders (n = 47)	Non-welders (n = 39)	p value
Age (year), Median (IQR)	40.00 (10.00)	39.00 (13.00)	41.00 (9.00)	0.183 ^**a**^
BMI (kg/m^2^), Mean ± SD	23.89 (2.76)	23.77 (2.70)	24.04 (2.86)	0.616 ^**b**^
Education level				0.550 ^**c**^
Primary school	29(33.7)	14 (29.8)	15 (38.5)	
Secondary school	22 (25.6)	14 (29.8)	8 (20.5)	
High school and above	35 (40.7)	19 (40.4)	16 (41.0)	
Marital status				0.889 ^**c**^
Single or divorce	28 (32.6)	15 (31.9)	13 (33.3)	
Married	58 (67.4)	32 (68.1)	26 (66.7)	
Income				0.370 ^**c**^
<15000	37 (43.0)	20 (42.6)	17 (43.6)	
15000–30000	43 (50.0)	22 (46.8)	21 (53.8)	
>30000	6 (7.0)	5 (10.6)	1 (2.6)	
Alcohol consumption				0.090 ^**c**^
Former/never drinker	24 (27.9)	17 (36.2)	7 (17.9)	
Current drinker	62 (72.1)	30 (63.8)	32 (82.1)	
Smoking status				0.284 ^**c**^
Former/never smoker	34 (39.5)	21 (44.7)	13 (33.3)	
Current smoker	52 (60.5)	26 (55.3)	26 (66.7)	
Mn (ug/g creatinine)	0.59 (1.13)	0.96 (1.04)	0.22 (0.98)	0.000 ^**a**^
Cr (ug/g creatinine)	0.50 (0.71)	0.63 (0.89)	0.22 (0.50)	0.003 ^**a**^
Pb (ug/g creatinine)	10.89 (8.67)	11.53 (9.51)	10.53 (7.56)	0.165 ^**a**^

^**a**^ Data was skewed distributed and presented as Median (Interquartile Range, IQR), Mann-Whitney U test was used for comparison of skewed distributed variables.

^**b**^ Data was normally distributed and presented as Mean ± SD, t- test was used for comparison.

^**c**^ Data was presented as Number (Percent, %), Chi-square test was used for categorical variables.

The urine metals level between the welder group and the non-welder group are presented in Table 1. Urine Mn level of the welder group (median 0.96 ug/g creatinine) was significantly higher than the non-welder group (median 0.22 ug/g creatinine, p < 0.001). For urine Cr level, the welder group (median 0.63 ug/g creatinine) was statistically higher than the non-welder group (median 0.22 ug/g creatinine, p < 0.01). There was no statistical difference for Pb level between the welder and non-welder groups (p > 0.05).

When all the workers were divided into low and high exposed groups by the median Mn level (0.59 ug/g creatinine), 66.0 percentage of welders were in high Mn-exposed group, while non-welders have 30.8 percentage in the high Mn-exposed group (p < 0.01) (Table 2). When divided by median Cr level (0.50 ug/g creatinine) into low and high exposed group, welders had 61.7 percentage in high Cr-exposed group, while 38.5 percentage of non-welder group were exposed in the high Cr-exposed group (p < 0.05). When the workers were divided by the median Pb level (10.89 ug/g creatinine), the distribution was not statistically different between welders and no-welder groups.

**Table 2:** Comparison of urine metals (Mn, Cr, and Pb) between occupation types and metal groups.

Occupation type	Mn level group (Median: 0.59 ug/g creatinine)			Cr level group (Median: 0.50 ug/g creatinine)			Pb level group (Median: 10.89 ug/g creatinine)		
	Low exposure	High exposure	p value	Low exposure	High exposure	P value	Low exposure	High exposure	p value
Welder	16 (34.0)	31 (66.0)	0.001	18 (38.3)	29 (61.7)	0.032	21 (44.7)	26 (55.3)	0.279
Non-welder	27 (69.2)	12 (30.8)		24 (61.5)	15 (38.5)		22 (56.4)	17 (43.6)	

Data were presented as Number (Percent, %), Chi-square test was used for categorical variables.

When all the workers were grouped by occupation types as shown in Table 3, the mean value of Hct in the welder group was 44.58 ± 2.84 vol%, significantly higher than the non-welder group (43.07 ± 3.31 vol%, p = 0.026). The mean value of Lymphocyte ratio % in the welder group (30.29 ± 6.19%) was lower than the non-welder group (32.32 ± 6.64%), but marginally significant (p = 0.085). When the workers were grouped by urine Mn level as shown in Table 3, the median value of WBC in the high Mn-exposed group (6.93 ± 1.59 X 10^6^ Cell/ml) was significantly lower than the low Mn-exposed group (7.90 ± 2.13 X 10^6^ Cell/ml, p = 0.018). No significant difference was found when blood parameters were grouped based on Pb or Cr level (all p > 0.05).

**Table 3:** Characteristics of hemostatic blood parameters stratified by occupation type and metal level group.

Variables	Occupation type			Mn level			Cr level			Pb level		
	Welder N = 47	Non-welder N = 39	p value	High-exposure N = 44	Low-exposure N = 44	p value	High-exposure N = 44	Low-exposure N = 44	p value	High-exposure N = 44	Low-exposure N = 44	p value
Hct Vol%	44.58 (2.84)	43.07 (3.31)	0.026 ^a^	43.63 (2.79)	44.16 (3.47)	0.436 ^a^	44.20 (3.15)	43.57 (3.13)	0.356 ^a^	44.31 (2.94)	43.47 (3.31)	0.212 ^a^
WBC X10^6^ Cell/ml	7.00 (2.70)	7.40 (2.10)	0.852 ^b^	6.93 (1.59)	7.90 (2.13)	0.018 ^a^	7.05 (2.25)	7.30 (2.33)	0.385 ^b^	7.40 (2.60)	7.10 (1.80)	0.829 ^b^
Neutrophil ratio %	55.76 (7.63)	54.98 (8.79)	0.661 ^a^	54.43 (7.89)	56.38 (8.35)	0.268 ^a^	56.04 (8.83)	54.74 (7.40)	0.461 ^a^	55.94 (8.28)	54.86 (8.06)	0.538 ^a^
Lymphocyte ratio %	30.29 (6.19)	32.32 (6.64)	0.085 ^a^	32.07 (5.97)	30.35 (6.81)	0.217 ^a^	31.53 (6.94)	30.86 (5.90)	0.630 ^a^	30.51 (6.48)	31.90 (9.20)	0.321 ^a^
Monocyte ratio %	7.60 (1.77)	7.98 (2.29)	0.409 ^a^	7.91 (1.91)	7.64 (2.13)	0.535 ^a^	7.79 (2.07)	7.76 (1.98)	0.943 ^a^	7.50 (3.40)	7.60 (2.20)	0.635 ^b^
Eosinophil ratio %	3.40 (4.70)	2.60 (3.40)	0.120 ^b^	3.00 (4.40)	3.10 (3.80)	0.890 ^b^	3.00 (4.07)	3.20 (4.55)	0.439 ^b^	3.40 (4.30)	2.50 (4.30)	0.090 ^b^

Normally distributed variables were presented as Mean ± SD. Skewed distributed variables were presented as Median (Interquartile Range, IQR).

^a^ t-test for comparison of normally distributed variables.

^b^ Mann-Whitney U test for comparison of skewed distributed variables.

Table 4 presents the correlations between urine metals level and Hemostatic blood parameters in welding fume exposed workers. The distribution for urine metal concentrations, WBC and Eosinophil ratio were skewed and these variables followed a normal distribution after logarithmic transformation. Pearson correlation was used to evaluate the correlation among log-transformed blood parameters and log-transformed metals (Cr, Mn and Pb). Positive low correlations were noted between urine Cr and urine Mn at log-transformed scales (*r*_*p*_ = 0.278, p < 0.01). Negative low correlation was noted between log-transformed Mn level and log-transformed WBC counts (*r*_*p*_ = - 0.279, p < 0.01). Furthermore, moderate to high correlations between Hemostatic Blood particles were also identified (p < 0.05).

**Table 4:** Pearson correlation analysis for log-transformed urine metals and blood parameters.

	Lg Mn	Lg Pb	Lg Hct	Lg WBC	Lg Neutrophil	Lg Lymphocyte	Lg Monocyte	Lg Eosinophil
Lg Cr	0.278**	0.202	0.077	- 0.121	0.051	0.040	- 0.004	- 0.096
Lg Mn		0.192	- 0.100	- 0.279**	- 0.115	0.134	0.109	- 0.007
Lg Pb			0.165	- 0.041	- 0.026	0.010	- 0.001	0.137
Lg Hct				0.241*	0.244*	- 0.267*	- 0.011	- 0.056
Lg WBC					0.480 **	- 0.563**	-0.464**	0.038
Lg Neutrophil						- 0.834**	-0.278*	- 0.427**
Lg Lymphocyte							0.263*	0.038
Lg Monocyte								- 0.219*

* p < 0.05.

** p < 0.01.

From the linear regression models shown in Tables 5 and 6, hemostatic blood particles were log-transformed and used as dependent variables for analyzing the associations between blood hemostatic parameters and welding fume exposure. All effects were adjusted by Age (years), body mass index (kg/m^2^), and smoking status in all workers. When taking occupation type as independent variables, increased Hct (vol%) was found in welders but it was marginally significant (β = 0.014, p = 0.055). If taking the high and low Mn-exposed exposure as independent variables, significantly decreased WBC was found in the high Mn exposure group (β = - 0.049, p = 0.045). Lymphocyte ratio was positively associated with Mn levels in all workers, but was not significant (β = 0.033, p = 0.131). No significant association was found when taking Pb or Cr as independent factors in the regression models (all p > 0.05).

**Table 5:** Effects of welding fume exposure on blood hemostatic parameters.

Factors	Hct Vol%			WBC x 10^6^ Cell/mm			Neutrophil ratio %		
	β	95% CI	p value	β	95% CI	p value	β	95% CI	p value
Occupation type: (non-welders as reference)	0.014	0.000 to 0.028	0.055	0.010	- 0.039 to 0.060	0.675	0.007	- 0.021 to 0.036	0.621
Cr (low group as reference)	0.008	- 0.006 to 0.022	0.242	- 0.028	- 0.076 to 0.020	0.244	0.009	- 0.019 to 0.037	0.507
Mn (low group as reference)	- 0.005	- 0.019 to 0.009	0.499	- 0.049	- 0.096 to -0.001	0.045	- 0.014	- 0.042 to 0.015	0.340
Pb (low group as reference)	0.009	- 0.005 to 0.024	0.206	0.015	- 0.035 to 0.065	0.554	0.009	- 0.020 to 0.038	0.548

Age (years), body mass index (kg/m^2^), and smoking status were adjusted as covariates.

Hemostatic blood particles were log-transformed.

**Table 6:** Effects of welding fume exposure on blood hemostatic parameters.

Factors	Lymphocyte ratio %			Monocyte ratio %			Eosinophil ratio %		
	β	95% CI	p value	β	95% CI	p value	β	95% CI	p value
Occupation type: (non-welders as reference)	-0.023	- 0.067 to 0.020	0.293	- 0.013	- 0.067 to 0.040	0.617	0.173	0.002 to 0.345	0.173
Cr (low group as reference)	0.000	- 0.043 to 0.043	0.992	0.000	- 0.053 to 0.052	0.985	- 0.083	- 0.255 to 0.088	0.337
Mn (low group as reference)	0.033	- 0.010 to 0.076	0.131	0.025	- 0.028 to 0.077	0.351	- 0.077	- 0.250 to 0.097	0.382
Pb (low group as reference)	-0.015	- 0.060 to 0.030	0.508	0.010	- 0.045 to 0.065	0.719	0.086	- 0.093 to 0.264	0.343

Age (years), body mass index (kg/m^2^), and smoking status were adjusted as covariates.

Hemostatic blood particles were log-transformed.

## Discussion

In recent years, there have been increasing number of researches focusing on the hematological blood variables to access the toxicity of welding fumes in humans and animals. In the present investigation, the weld processes and mild metal type may result in exposure to high concentrations of PM with metals of Fe, Mn, and Cr. We focused on the effects of the urine metal level on the hematological variables after a 3 weeks exposure cessation. Our participants (both welders and non-welders) were working in the same confined space and all the welders were extensively involved in mild welding work. Of all the workers, welders were provided with professional helmet and respirators (respirator film 3M 2091 P3-BR), while other workers, such as fitter, fire watch, and the other workers used masks and cloth hats to cover their mouth and head. Thus, we divided the workers based on the occupation type into welders and non-welders as welders were supposed to be exposed to high concentrations of welding fumes, particulate matter, and toxic metals. Some other workers working in the confined space like fitters, who always worked together with the welders to assemble and install steel structure, also had higher chance to be exposed to the welding fumes working without breathing respirators. Besides, all the workers had different habits of using PPEs to protect fumes and particles. Therefore, the workers were also divided into high and low exposure groups based on the Mn, Cr and Pb levels in urine, as well as based on their occupation types as welders and non-welders.

Our study included “healthy” samples of 49 welders and 37 non-welders, which was aimed to conservatively estimate the health effects of welding fumes. Obesity workers with BMI > 28 (20 people), self-reported a history of Diabetes, Hyperlipidemia and Metabolic syndrome (11 people), or self-reported use of hypertension and CVDs medication (21 people) were all excluded, and there were 123 eligible respondents—were recruited in this present study at the study starting point and 86 people stayed in the follow up. 27 people lost follow up and 8 people’s questionnaire were incomplete and were cancelled. Another 2 people were excluded since their WBC counts were abnormally high which might be caused by the urge inflammation. It must be mentioned that such a strict inclusion criterion would exclude the high CVD risks person caused by long term welding exposure, however, considering the relatively young age 20 to 50, such a strict inclusion criterion would reduce the interference of the familial inheritance of CVD risks. By doing so, we hope the confounding effects of the familial inheritance of CVD disease would be minimized to the greatest extent.

The bare steel of the plates (the storage tank) and heavy-duty steel structures were mainly made of carbon-manganese-silicon steel plate A573, which contained Carbon max (0.23%), Mn (0.85–1.2%), Phosphorus max (0.030%), Sulfur max (0.030%) and Silicon (0.15–0.40%). The chemical composition of deposited metal Electrodes (KOBELCO) contained Mn (≤1.60%), Si (≤0.75%), P (≤0.040%), Cr (≤0.20%), Ni and Mo (≤0.30%). In our investigation, compared to non-welders, welders were had about 3-to-4-fold higher urine Mn and Cr concentrations, but not as Pb. The results suggested that welding process type (FCAW and SAW) and mild steel plate may lead to higher amounts of Mn and Cr exposure in the urine. Some other studies also reported that welding in confined space was associated with a higher Mn concentration and FCAW was related to 4.47 higher Mn concentrations compared to Gas metal arc welding with solid wire [24]. In the present study, urine Mn and urine Cr were positively correlated (*r*_*p*_ = 0.278, p < 0.01). There were significant correlations between WBC and urine Mn, but no significant correlations between blood parameters and Pb or Cr level groups (all p > 0.05), even Cr and Mn were positively correlated (*r*_*p*_ = 0.278, p < 0.01). The above results may suggest that in the FCAW and SAW welding process with mild steel, Mn was supposed to play a key role in the hemodynamic balance relating to pathogenesis of welding fume toxic.

Blood parameters are considered as physiological indicators of the whole-body functioning and therefore were important in diagnosing the structural and functional status of the humans exposed to toxicants [7,28]. Compared to the non-welders, welders showed a slight increase in Hct vol% (p = 0.026), but the other blood indices such as Lymphocyte ratio % and WBC were not significant. In addition, no significant positive relationship between WBC counts and occupation type was found in this study in the linear regression analysis adjusted by variables or without adjustment. This might be due to one third of the non-welders being exposed to the welding fumes working without breathing respirators, which made the relationship insignificant. When all the workers were divided into high and low exposure groups based on urine Mn, Cr and Pb levels, the results of generalized linear models showed that log transformed WBC was negatively associated with external exposure of Mn level groups (β = - 0.049, p = 0.045). All effects were adjusted by age, BMI, and smoking. When taking continuous urine Mn level as independent variables, log transformed WBC was negatively associated with external exposure of Mn levels (β = - 0.050, p = 0.031, data as not shown in the tables) after adjustment. These results were inconsistent with some of the reports about the effects of welding fumes on hematological parameters.

Like the mechanisms that link PM 2.5 to CVDs, the hypothetical mechanisms of welding fume toxicity center on oxidative stress and inflammatory. Various pro-oxidant, inflammatory and/or hemodynamic agents as the metal-related, ROS-mediated consequences may trigger a range of reactions in the vascular system. In a cohort study assessing the life-long health risks of occupational welders [3] and the effects on WBC counts, the welders reported to have higher levels of WBC counts, and higher CVD risks [29]. The popular mechanisms on toxicity of welding fumes were in agreement that PM was associated with increased WBC and neutrophil counts [28], and increased WBC counts lead to higher CVD and CHD risks [19,30]. Regarding welding fume, some studies have shown that short-term or long-term exposure to air pollutants may affect hemodynamic balance, but the results were not consistent. Some findings on animals were in accordance with this mechanism which also reported an increase in WBC count after exposure with Mn compounds in rats and dogs [31,32]. In contrast, some studies have reported a decrease in WBC count after Mn exposure in rats [33]. There were also reports with inconsistent results among humans [17,34,35]. Kim, J.Y., et al. reported that high levels of welding fume exposure induce acute systemic inflammation in a relatively young, healthy working population with a significant increase in WBC and neutrophil counts, neutrophils, fibrinogen after 6 hours of exposure, but 24 hours post-baseline changes were not significant [17]. Another study that focused on Heart Rate Variability (HRV), hemostatic and acute inflammatory blood parameters in healthy adults (non-welders) after a short-term high-level exposure to welding fumes reported no immediate effects of clinical significance on blood hemostatic and acute inflammatory parameters after 5 hours in healthy subjects [35]. In a recently published study on workers exposed to Mn, the high-exposure group had a significantly higher level of MONO ratio and lower level of plasma complement C3, while no significant result was reported for WBC counts [36]. Given the great complexity of the immune cell repertoires and great time difference in renewal cycle of each cell repertoires (days to weeks) [37], there was not many studies focusing time series about Metals-induced hematological toxicity and possible mechanism behind the renewal cycle for immune cells. Literatures have shown that gene regulator may govern the self-renewal of haematopoietic stem cells in the renewal cycle. Another evidence to support that an uptake of particles into endothelial cells destroys lysosomal equilibrium which results in an imbalance between autophagosomes and autolysosomes [38]. These processes can obstruct waste degradation and delay the renewal of endothelial cells [38]. Most previous studies regarding welding fume exposure population focused on acute response after 3 hours, 8 hours or 24 hours after the exposure in humans or animals. In contrast, some studies only compared welders and controls to study lifelong risks of being welders without a clear declaration of time window of response, neither the welding techniques nor the welding fume dose. The results were not consistent and the discrepancy in the results might be due to different exposure conditions and different route of administration making it difficult to make direct comparison.

The hematopoiesis system redistributed and recovered with the process of Mn excretion from the body since Mn has a half-life of 10–42 days [39]. In the present study, after 3 weeks of cessation of Mn exposure, all altered hematological parameters might have been in the process of recovery. In a recent study about effects of welding fumes on hematological parameters of adult male Wistar rats [40], it was found that WBC count showed a significant increase in high dose Mn-exposed rats when compared with that of the control group, and they reported that after 60 days of cessation of MnCl_2_ exposure. All these altered hematological parameters showed recovery effects indicating resumption of normal functioning of hematopoietic tissues. In recovery group, there was a significant increase in Hct values and a reduction in WBC count when compared with all three MnCl_2_ treated groups in the adult male Wistar rats [40] while the values of these parameters in recovery group were almost comparable to that of the values in control group. The results of hematological differences in the Wistar rats and among the workers in the present study may indicate an activation and recovery of the immune system in response to tissue damage caused by toxicants. Our observations did not exclude the different outcomes as found by other authors and the possibility of the further appearance of alterations in these hematological parameters.

This study also had some limitations. The study was cross-sectional, measurements of both metal level and blood markers of effect were performed only once. Therefore, the estimated effect of exposure to welding fumes was rather uncertain. Non-welder group appears to lose follow up more than welder-group which might have an impact on overestimate or underestimate of the study results. The measurement of urine metal level was performed only once on each participant and then used for dividing workers into groups. Since measurements of blood markers were performed only once in the field study, it was difficult to make a clear declaration of time courses of response.

Unlike the other studies, we tried to use occupation type, as well as the metal level group as independent variables to assess the associations between welding fume exposure and blood hematological response. Significant findings on WBC counts between different Mn level groups were reported but not as for occupation type. This is probably because, working in the confined space without PPEs, non-welders may have high risks for breathing in higher fume concentrations. To confirm a casual relationship, healthy control group without welding fume exposure may include as another comparison group for future study. This was reported and discussed with the HSE department of the company and agreement was made regarding the necessity of PPEs for non-welder occupation types. Under industrial conditions, one preventive measure against excessive Mn retention is the periodical rotation of the workers in exposed positions, which is also important for the other relevant occupations.

Based on the present study, it can be concluded that welding fumes exposure showed an adverse impact on hematological parameters in workers. Mn was supposed to play a key role in the hemodynamic balance relating to pathogenesis of welding fume toxic. Therefore, further studies are suggested to better understand the mechanism(s) of Metals-induced hematological toxicity, the time window and possible mechanism behind the recovery.

## Supporting information

S1 File Content validity of the questionnaire. (PDF)

S2 File Questionnaire in Thai and English. (PDF)

S3 File Manuscript data set PLOSone. (XLS)
