# Supplementary material for: Associations between welding fume exposure and blood hemostatic parameters among workers exposed to welding fumes in confined space in Chonburi, Thailand
Source: PLoS One. 2021 Nov 18;16(11):e0260065. doi: 10.1371/journal.pone.0260065 (PMC8601467; doi:10.1371/journal.pone.0260065)
Supplement: S1 File — (PDF) [file pone.0260065.s001.pdf]

Ning Li

6079178953

Index of Item Objective Congruence (IOC)

The Test for Validity of Research Questionnaire

Topic: Effect of Occupational Welding Fume Exposure on Hemostatic & Inflammatory Blood Parameters and Cardiovascular Risks in Pipeline Welders: A Longitudinal Study

Name of Researcher: Ning Li

Student ID: 6079178953

Position: Doctorate Degree student of Public Health Science

Result and test score: ………0.81…………………………………………………………………….

Recommendation: ……………see comments in detail on next page ……………………

……………………………………………………………………………………………………

…………………………………………………………………………………………………..

……………………………………………………………………………………………………

Name………Ning Li…………..……….

Signature……………………

Date………………………….

Summary of the IOC feedback

**Evaluation list (**Total questions: 32**)**	**Respondent Information**			
	Arjan. Wattasit	Arjan. Ratana	Mr.Xu guang Zhang	Average
	25/32	25/32	28/32	
Score	0.78	0.78	0.88	0.81
General information				
	Arjan. Wattasit	Arjan. Ratan a	Mr.Xu guang Zhang	Result
1. Title or working position…………………..……………	1	1	1	
2. Birth of date: Year……… Month………Date………	1	1	1	
3. Marital status:<br>☐ Single<br>☐ Married<br>☐ Widowed<br>☐ Divorced	1	1	1	
4. Time of last health check:<br>Year……. Month……. Date………	1	1	1	
5. Height (cm)………… Weight (kg)………….	1	1	1	
6. Highest education level:<br>☐ No education<br>☐ Primary school<br>☐ Secondary school<br>☐ High school<br>☐ Bachelor<br>☐ Master and above	1	1	1	
7. Income<br>☐ < 15,000 Bath	1	1	1	

☐ 15,000 – 30,000 Bath<br>☐ 30,000 - 45,000 Bath<br>☐ 45,000 - 70,000 Bath<br>☐ > 70,000 Bath				
Health history				
1. Do you have, or had, any of the following? Please fill in major health issues diagnosed by the doctors:<br>☐ Diabetes<br>☐ Hypertension<br>☐ Hypercholesterolemia,<br>☐ Coronary heart disease (Angina, MI), Cerebral thrombosis and Other CVD diseases<br>☐ Cancer<br>☐ Asthma<br>☐ Others, please specify ………………	0	1	1	
2. Are you currently taking any medically prescribed, or self-prescribed medications? If any, please list what are the medicines about:<br>☐ Diabetes<br>☐ Hypertension<br>☐ Hypercholesterolemia,<br>☐ Coronary heart disease (Angina, MI), Cerebral thrombosis and Other CVD diseases<br>☐ Cancer<br>☐ Asthma<br>☐ Others, please specify ………………	0	1	1	
3. Do your Mother/Father/Siblings have, or had, any of the following health problems? Please fill in major health issues diagnosed by the doctors:<br>☐ Diabetes<br>☐ Hypertension<br>☐ Hypercholesterolemia,<br>☐ Coronary heart disease (Angina, MI), Cerebral thrombosis and Other CVD diseases<br>☐ Cancer<br>☐ Asthma	1	1	1	

☐ Others, please specify ………………				
**Life style in last year**				
Sports:				
1. Do you exercise (have a body activity continuously for at least 30 minutes)?<br>☐ No (please pass the following questions)<br>☐ Yes, about ……. times per week.	1	1	1	
2. What kind of physical activity do you usually do? (Please check all that apply)<br>☐ Walking<br>☐ Jogging<br>☐ Biking<br>☐ Swimming<br>☐ Free weights<br>☐ Strength machines<br>☐ Yoga or Pilates	0	1	1	
Diet:				
1. What is your diet habits (choose the place where you eat)?<br>☐ Cook and eat home<br>☐ Canteen<br>☐ Snack (take away or fast food)<br>☐ Restaurant	1	1	1	
2. What kind of taste do you prefer?<br>☐ Light<br>☐ Salty<br>☐ Greasy<br>☐ Sweat	1	-1	1	Change

☐ Spicy<br>* ***What kind of taste do you prefer? (You can choose more than one)***				
3. Do you often have fried or roasted food?<br>☐ Often, 5-7 days per week<br>☐ Sometimes, 3-4 days per week<br>☐ Occasionally,1-2 days per week<br>☐ Seldom, less than 1 time per week<br>☐ No	1	1	1	
Smoke & Alcohol:				
1. Do you smoke? How many cigarettes do you smoke every day? And how long have you been smoking?<br>☐ Never ever<br>☐ Currently smoke, about ……. cigarettes per day<br>Have lasted for ……. Years<br>☐ Used to smoke, lasted for ……. Years	1	1	1	
2. What is the frequency of second hand smoke exposure per week on average?<br>☐ Every day<br>☐ 4-6 days per week<br>☐ 1-3 days per week<br>☐ Never	1	1	1	
3. Do you drink alcohol? How often do you drink?<br>☐ Never ever<br>☐ Currently drink, about ……. times per week,<br>have lasted for …… years<br>☐ Used to drink, lasted for ……. years	0	1	1	
Sleep:				
1. How many hours do you sleep each night?<br>☐ > 8h<br>☐ 6-8h<br>☐ 4-6h<br>☐ <4h	1	1	1	

☐ Unsure				
2. Do you have trouble in sleeping?<br>☐ No<br>☐ Difficulty falling asleep<br>☐ Often wake up early<br>☐ Often wake up half way<br>☐ light sleep, dreaminess	1	1	1	
3. How long have you been with the sleeping troubles?<br>☐ No sleeping problems<br>☐ Just start<br>☐ < 1 yrs.<br>☐ 1-5 yrs.<br>☐ more than 6 years<br>*If you sleeping troubles, how long have you been with the sleeping troubles? (if no, please skip)*<br>*……………. years …………. months*	1	-1	-1	Change
4. Do you take sleeping medicine?<br>☐ No<br>☐ Irregularly<br>☐ Often<br>☐ Everyday<br>☐ In special treatment	1	1	1	
Welding fume exposure conditions				
**<u>Question 1 only for non-workers:</u>**<br>1. As office workers, do you have any chance to exposure to welding fumes longer than 10 minutes, within 2 meters from the welding source?<br>☐ Often, more than 15 days per month<br>☐ Sometimes, about 10 – 15 days per month<br>☐ Occasionally, 5-10 days per month<br>☐ Seldom, about 1-5 days per month<br>☐ No, no chance to exposure	1	1	1	
**<u>Questions 2-6 for welders:</u>**				
2. How long (how many years) have you been working a welder?	1	1	1	

Please specify the number of years:<br>………………years……….months				
3. In general, during the past years working as welders, how many months did you take the welding tasks per year?<br>Please specify the number of months:<br>………………months/year	1	1	1	
4. In general, during the past years working as welders, how many days did you take the welding tasks per month?<br>Please specify the number of hours per week:<br>………………days/month	1	1	1	
5. In general, during the past years, how many hours did you take the welding tasks per day?<br>Please specify the number of hours per day:<br>………………hours/day	1	1	1	
6. How often do you use shield and respirator for protecting welding fumes during doing welding tasks?<br>☐ ***Every time, 100% time***<br>☐ ***Always, > 80 % time***<br>☐ ***More than half time, 50-80 % time***<br>☐ ***Less than half time, 20-50 % time***<br>☐ ***Seldom use, < 20 % time***	1	1	1	Edit<br>Add %
**Questions 7-9 for welders:**				
7. How many night shifts do you have per week?<br>☐ None<br>☐ Once a week<br>☐ Twice per week<br>☐ 3 times per week<br>☐ > 3 times per week	0	1	-1	It is relating to CVDs, so this question is kept.
8. Are you stressed, or feel comfortable on your work?<br>☐ Yes<br>☐ Sometimes	0	-1	1	Change

☐ Seldom<br>☐ Unclear<br>☐ No<br>** Are you stressed on your work?*				
9. What kind of welding type you always use? (you can choose more than two)<br>☐ Stainless steel (SS) welding<br>☐ Mild steel (MS) welding<br>☐ Mixed type<br>☐ Not sure<br>☐ Others<br>**** what kind of welding type did you always use in the past year? (you can choose more than one).***	0	0	1	change
10. How often do you take part in relative training education programs hold by the company? Such as about welding technical skills or safety & health effects about welding process?<br>☐ About 5 or more than 5 times per year<br>☐ About 3 or 4 times a year<br>☐ About 1 or 2 times a year<br>☐ None	1	1	1	
