# Supplementary material for: Associations between welding fume exposure and blood hemostatic parameters among workers exposed to welding fumes in confined space in Chonburi, Thailand
Source: PLoS One. 2021 Nov 18;16(11):e0260065. doi: 10.1371/journal.pone.0260065 (PMC8601467; doi:10.1371/journal.pone.0260065)
Supplement: S2 File — (PDF) [file pone.0260065.s002.pdf]

Employee Health Questionnaire

แบบสอบถามสำหรับกลุ่มตัวอย่าง

1. General information ข้อมูลทั่วไป

**1. General Information**	
Title or working position<br>**ภาระงานที่ทำ ตำแหน่งงานวัน**:	Height:................ Weight:.....................
**Date of Birth**:<br>**วันเกิด: .... /D วัน .... /M เดือน ..../Y ปี**	Marital status: **สถานภาพการสมรส**<br>☐ **โสด** single ☐ **แต่งงาน** marry ☐ **หย่า** divorced
Income per month<br>**(รายได้ ต่อ เดือน)**<br>☐ < 15,000 Bath<br>☐ 15,000 – 30,000 Bath<br>☐ 30,000 - 45,000 Bath<br>☐ 45,000 - 70,000 Bath<br>☐ > 70,000 Bath	Highest education level การศึกษาขั้นสูงสุด:<br>☐ No education ไม่ได้เรียน<br>☐ Primary school ประถม<br>☐ Secondary school มัธยมต้น<br>☐ High school มัธยมปลาย<br>☐ Bachelor ปริญญาตรี<br>☐ Master and above ปริญญาโท หรือมากกว่า

2. HEALTH HISTORY คำถามเกี่ยวกับสุขภาพ

**Health history คำถามเกี่ยวกับ "สุขภาพ"**
1. Do you have, or had, any of the following? Please fill in major health issues diagnosed by the doctors:<br>คุณมีโรคประจำตัวไหม (โดยผ่านการวินิจฉัยจากแพทย์) ....<br>คุณมีหรือเคยเป็นโรคดังต่อไปนี้หรือไม่?((โดยผ่านการวินิจฉัยจากแพทย์))<br>☐ Diabetes เบาหวาน<br>☐ Hypertension ความดันสูง<br>☐ Hypercholesterolemia ไขมันในเลือดสูง)<br>☐ Coronary heart disease (Angina, MI), Cerebral thrombosis and Other CVD diseases โรคหลอดเลือดหัวใจ (Angina, MI), การเกิดลิ่มเลือดในสมองและโรค CVD อื่น ๆ (โรคหัวใจ)<br>☐ Cancer โรคมะเร็ง<br>☐ Asthma โรคหอบหืด<br>☐ Others, please specify อื่น ๆ (โปรดระบุ)...<br>☐ No ไม่
2. Are you currently eating any regular medications? (If there is, please specify the drug-related list)<br>ในปัจจุบันคุณกำลังกินยาประจำ อะไรไหม? (ถ้ามีโปรดระบุรายการยาที่เกี่ยวกับ)

☐ no ไม่มี

☐ yes มี ……………………………………………………………………………(please specify โปรดระบุ)

3. Do your Mother/Father/Siblings have, or had, any of the following health problems? Please fill in major health issues diagnosed by the doctors:

ประวัติครอบครัวคุณ (เช่น พ่อ, แม่ หรือเครือญาติ) มีโรคเหล่านี้ไหม?

☐ Diabetes เบาหวาน

☐ Hypertension ความดันสูง

☐ Hypercholesterolemia ไขมันในเลือดสูง)

☐ Coronary heart disease (Angina, MI), Cerebral thrombosis and Other CVD diseases โรคหลอดเลือดหัวใจ (Angina, MI), การเกิดลิ่มเลือดในสมองและโรค CVD อื่น ๆ (โรคหัวใจ)

☐ Cancer โรคมะเร็ง

☐ Asthma โรคหอบหืด

☐ Others, please specify อื่น ๆ (โปรดระบุ)……………………………………….

☐ No ไม่

3. LIFE STYLE in last year ประวัติกิจกรรมของท่านก่อนหน้านี้

Sport คำถามเกี่ยวกับ "ด้านกีฬา"

1. Do you exercise after finishing your work? (have a body activity continuously for at least 30 minutes)

คุณออกกำลังกายหลังจากทำงานเสร็จหรือไม่? (ประมาณ 30 นาที / ครั้ง)

☐ No (please pass the following questions) ไม่เลย (ข้ามคำถามต่อไปนี้)

☐ Yes, about ……. times per week. เคย (ประเภทกีฬา……………………………… ครั้ง …………….. ต่อสัปดาห์)

2. What kind of physical activity do you usually do? (Please check all that apply to you).

คุณออกกำลังกายแบบไหน? (โปรดเลือกทุกข้อที่ตรงกับคุณ) ………

☐ Walking เดิน

☐ Jogging วิ่ง

☐ Biking ปั่นจักยาน

☐ Swimming ว่ายน้ำ

☐ Free weights Strength machines ยกน้ำหนัก

☐ Yoga or Pilates โยคะ

☐ No ไม่เลย

Diet: คำถามเกี่ยวกับ "การกิน"

3. What are your diet habits or where do you often eat? (choose the place where you eat) ……..

คุณรับประทานอาหารแบบไหน, หรือคุณมักจะกินที่ไหน? (เลือกตามสถานที่ที่คุณกิน)

☐ Cook and eat home ทำเองและกินที่บ้าน

☐ Canteenโรงอาหารในที่ทำงาน

☐ Buy cooked food at the open market ซื้อกับข้าวในตลาด

☐ Snack (take away or fast food) อาหารจานด่วน

☐ Restaurant ร้านอาหาร

4. What kind of taste do you prefer (you can choose more than 1)?

☐ Light ธรรมดา

☐ Salty รสเค็ม

☐ Greasy อาหารเลี่ยน

☐ Sweat รสหวาน

☐ Spicy รสจัด

5. Do you often have fried or roasted food?

คุณรับประทานอาหารประเภททอดหรือคั่ว บ่อยแค่ไหน

☐ Often, 5-7 days per week บ่อยครั้ง, 5-7 วัน/สัปดาห์

☐ Sometimes, 3-4 days per week บางครั้ง, 3-4 วัน/สัปดาห์

☐ Occasionally,1-2 days per week ครั้งคราว, 1-2 วัน/สัปดาห์

☐ Seldom, less than 1 time per week น้อยมาก, <1 วัน/สัปดาห์

☐ No ไม่รับประทาน

Smoke & Alcohol คำถามเกี่ยวกับ **“การสูบบุรี่” และ “ดื่มแอลกอฮอร์”**

6. Do you smoke? How many cigarettes do you smoke every day? And how long have you been smoking?

คุณสูบบุหรี่หรือไม่ ? คุณสูบบุหรี่กี่มวนต่อวัน ? คุณสูบหรี่มานานแค่ไหน ?

☐ Never ever ไม่เคย

☐ Currently smoke, about ……. cigarettes per day, have lasted for ……. Years
ปัจจุบันสูบบุหรี่ ประมาณ …………. มวน / วัน สูบบุหรี่มานานกี่ ………ปี (years)…………เดือน (month)

☐ Used to smoke, lasted for ……. Years
เคยสูบบุหรี่ . ………ปี (years)…………เดือน (month)

7. What is the frequency of smoking fume exposure per week on average?

คุณสัมผัส “ควันบุหรี่จากมือสอง” บ่อยแค่ไหน

☐ Every day ทุกวัน

☐ 4-6 days per week 4-6 วัน / สัปดาห์

☐ 1-3 days per week 1-3 วัน / สัปดาห์

☐ Never ไม่เคยสัมผัสควันบุหรี่

8. Do you drink? How often do you drink? คุณดื่มแอลกอฮอร์บ่อยไหม

☐ Never ever ไม่เคย

☐ Currently drink, about ……. times per week, have lasted for …... years
ปัจจุบันดื่ม ประมาณ …………. มวน / วัน ดื่มมานานกี่ ………ปี (years)

☐ Used to drink, lasted for ……. Years
เคยดื่ม . ………ปี (years)

Sleep คำถามเกี่ยวกับ “การนอน”

9. Sleeping hours each night: นอนกี่ชั่วโมง / คืน

☐ >8h >8 ชั่วโมง / คืน

☐ 6-8h 6-8 ชั่วโมง / คืน

☐ 4-6h 4-6 ชั่วโมง / คืน

☐ < 4h <4 ชั่วโมง / คืน

☐ Unsure ไม่แน่ใจ

10. Do you have trouble in sleeping?

คุณมีปัญหาด้านการนอนหลับไหม

☐ No ไม่มีปัญหา

☐ Difficulty falling asleep ยากที่จะนอนหลับ

☐ Often wake up early บ่อยครั้ง ตื่นเร็ว

☐ Often wake up half way บ่อยครั้ง ตื่นกลางดึก

11. If you sleeping troubles, how long have you been with the sleeping troubles? (if no, please skip)

คุณนอนไม่ค่อย หลับมานานแค่ไหน (ถ้าไม่ ข้ามไปทำข้อต่อไป)

☐ …………year ปี …………..month เดือน

☐ no problem ไม่มีปัญหา ลับสบายดี

12. Do you take sleeping medicine? คุณกินยานอนหลับไหม

☐ No ไม่เคย

☐ Irregularly กินไม่สม่ำเสมอ, แล้วแต่เหตุการณ์

☐ Often กินบ่อยครั้ง

☐ Everyday กินทุกวัน

☐ In special treatment กำลังรับการรักษา

4. Welding fume exposure conditions

For non-welders, please answer: กรณีพนักงาน "คนที่ไม่ใช่ช่างเชื่อม"
1. As not workers, do you have any chance to exposure to welding fumes longer than 5 minutes, within 2 meters from the welding source?<br>ในขณะทำงาน คุณได้สัมผัสควันจากการแหล่งเชื่อม มากกว่า 15 นาที ในช่วงระยะห่าง 2 เมตรหรือไม่?<br>☐ Often, more than 15 days per month บ่อยครั้ง, มากกว่า 15 วัน/เดือน<br>☐ Sometimes, about 10 – 15 days per month บางครั้ง, 10-15 วัน/เดือน<br>☐ Occasionally, 5-10 days per month ครั้งคราว, 5-10 วัน/เดือน<br>☐ Seldom, about 1-5 days per month น้อยมาก, 1-5 วัน/เดือน<br>☐ No, no chance to exposure ไม่ได้สัมผัส
2. Before working here Have you ever been exposed to welding fumes before?<br>ก่อนทำงานที่นี่ คุณเคยสัมผัสกับควันเชื่อมมาก่อนหรือไม่?<br>☐ no ไม่เลย<br>☐ เคย (ระยะเวลาที่สัมผัสประมาณ ....เดือน.............. ปี) used to (exposed for approximately ....month.............. Year)
3. How many night shifts do you have to work per week?<br>โดยปกติคุณทำงานช่วง "กะกลางคืน" กี่ครั้ง / สัปดาห์<br>☐ no ไม่เคย<br>☐ one time per week 1 ครั้ง/สัปดาห์<br>☐ two times per week 2 ครั้ง/สัปดาห์<br>☐ three times per week 3 ครั้ง/สัปดาห์<br>☐ more than 3 times per week >3 ครั้ง/สัปดาห์
4. What kind of prevention measures do you use to prevent dust or welding fumes during work?<br>มีวิธีการใดบ้างที่ใช้ป้องกันฝุ่นหรือควันเชื่อมในระหว่างการทำงาน?<br>☐ Not protection measures ไม่ได้ป้องกัน<br>☐ use masks to cover nose หน้ากากปิดจมูก<br>☐ Wear a hat that protects against inhalation of dust or fumes สวมหมวกที่ป้องกันการหายใจเอาฝุ่นหรือควันเข้าไปได้<br>☐ Use a head covering cloth to cover mouth and head ใช้ผ้าคลุมศีรษะ
5. Are you stressed or worried about the exposure to welding fumes in your workplace?<br>คุณเครียดหรือกังวลเกี่ยวกับการสัมผัสควันที่เกิดจากการเชื่อมในที่ทำงานของคุณหรือไม่?<br>☐ Very worried กังวลมาก<br>☐ A little worried กังวลเล็กน้อย<br>☐ Don't worry ไม่ต้องกังวล<br>☐ Not sure ไม่แน่ใจ

5. การสัมผัสควันจากการเชื่อม

If you are welders, please answer: (กรณีพนักงาน “ช่างเชื่อม” โปรดระบุ)	
1. How long (how many years) have you been working as a welder?<br>คุณเป็นช่างเชื่อมมานานเท่าใด	Please specify the number of years<br>…………………………..year<br>………………………….. ปี
2. In general, during the past years working as welders, how many months do you take the welding tasks per year?<br>โดยทั่วไปในช่วงปีที่ผ่านมา คุณทำงานเป็นช่างเชื่อมกี่เดือนต่อปี?	Please specify the number of days per week: ………………days/month<br>……………………….เดือน / ปี
3. In general, during the past years working as welders, how many days do you take the welding tasks per month?<br>โดยทั่วไปในช่วงปีที่ผ่านมา คุณทำงานเป็นช่างเชื่อมกี่วันต่อเดือน?	Please specify the number of days per week: ………………days/month<br>……………………….วัน / เดือน
4. In general, during the past years working as welders, how many hours do you take the welding tasks per day?<br>โดยทั่วไปในช่วงปีที่ผ่านมา คุณทำงานเป็นช่างเชื่อมกี่ชั่วโมงต่อวัน?	Please specify the number of hours per day: ………………hours/day<br>………………………. ชั่วโมง / วัน
5. How often do you use shield and respirator for protecting welding fumes during doing welding tasks?<br>คุณใช้ “โล่และเครื่องช่วยหายใจ”ในการป้องกันควันจากการเชื่อมในระหว่างการทำงานเชื่อมบ่อยแค่ไหน?<br>☐ Every time, 100% time ทุกครั้ง (100%)<br>☐ > 80 % time มากกว่า 80% ของเวลา ทำงาน<br>☐ More than half time, 50-80% time มากกว่าครึ่งเวลา, 50-80% ของเวลาทำงาน<br>☐ Less than half time, 20-50% time เวลาน้อยกว่าครึ่ง, 20-50% เวลาทำงาน<br>☐ Seldom use, < 20% time ใช้น้อยครั้ง น้อยกว่า 20%เวลาทำงาน	
6. Are you stressed or worried about the exposure to welding fumes in your workplace?<br>คุณเครียดหรือกังวลเกี่ยวกับการสัมผัสควันที่เกิดจากการเชื่อมในที่ทำงานของคุณหรือไม่?<br>☐ Very worried กังวลมาก<br>☐ A little worried กังวลเล็กน้อย<br>☐ Don't worry ไม่ต้องกังวล<br>☐ Not sure ไม่แน่ใจ	
7. How many night shifts do you have to work per week?<br>โดยปกติคุณทำงานช่วง “กะกลางคืน” กี่ครั้ง / สัปดาห์	

☐ no ไม่เคย

☐ one time per week 1 ครั้ง/สัปดาห์

☐ two times per week 2 ครั้ง/สัปดาห์

☐ three times per week 3 ครั้ง/สัปดาห์

☐ more than 3 times per week >3 ครั้ง/สัปดาห์

8. What kind of welding process do you always use? (You can choose more than one)

คุณใช้กระบวนการเชื่อมแบบใดเสมอ? (คุณสามารถเลือกได้มากกว่าหนึ่ง)

☐ Submerged-Arc Welding (SAW) การเชื่อมแบบอาร์คจมอยู่ใต้น้ำ (SAW)

☐ Flux Core-Arc Welding (FCAW) กระบวนการเชื่อมแบบฟลักซ์คอร์ (FCAW)

☐ Gas Tungsten-Arc Welding (GTAW) แก๊สเชื่อมทังสเตนอาร์ค (GTAW)

☐ mixed type ประเภทผสม

☐ not sure ไม่แน่ใจ

9. What kind of material do you use for welding? คุณใช้วัสดุอะไรเป็นหลักในการเชื่อม?

☐ carbon steel เหล็กกล้าคาร์บอน

☐ Stainless สแตนเลสเหล็ก

☐ Alloy โลหะผสม

☐ mixed type ประเภทแตกต่างกันไป

☐ not sure ไม่แน่ใจ

10. How often do you take part in relative training education programs? Such as trainings about safety rules?

คุณได้อบรม "กฎความปลอดภัยเกี่ยวกับการเชื่อม "บ่อยแค่ไหน

☐ more than 5 times per year ≥ 5 ครั้ง / ปี

☐ 3 to 4 times a year 3-4 ครั้ง / ปี

☐ 1 to 2 times a year 1-2 ครั้ง / ปี

☐ No have ไม่เคย
